# Egyptian General Population Knowledge and Awareness Toward Oral Cancer: A Cross‐Sectional Study

**DOI:** 10.1155/tswj/4032372

**Published:** 2026-03-16

**Authors:** Layla Hafed, Ahmed Abodowia, Mahmoud Elshazly, Mansour El Sayyed, Mariam Elshobaky, Menna-Allah Fayze, Mina Raafat, Gamilah Al-Qadhi

**Affiliations:** ^1^ Department of Oral Medicine and Diagnostic Science, Faculty of Dentistry, Saba University, Sana′a, Yemen; ^2^ Department of Oral and Maxillofacial Pathology, Faculty of Oral and Dental Medicine, Ahram Canadian University, Giza, Egypt, acu.edu.eg; ^3^ Internship Program, Faculty of Oral and Dental Medicine, Ahram Canadian University, Giza, Egypt, acu.edu.eg; ^4^ Department of Basic Dental Sciences, Faculty of Dentistry, University of Science and Technology, Aden, Yemen, hust.edu.vn

**Keywords:** awareness, Egypt, knowledge, oral cancer, risk factors

## Abstract

**Background:**

The present study aims to provide a thorough assessment of the level of knowledge and awareness of oral cancer among a general population of Egyptians and at correlating the main risk factors associated with the level of knowledge.

**Methods:**

One thousand twenty‐eight questionnaires were distributed to the general public at 10 public places located in Cairo, Egypt. Questionnaire results were summarized via descriptive statistics followed by correlation which was performed on the answers to different questions.

**Results:**

The overall knowledge of oral cancer among a population of Egyptian public was not strong, with 40% scoring 6–8 out of the maximum score 17. Only 14% of the participants acquired their knowledge from a healthcare provider. However, 47.2% did not know the symptoms of oral cancer. By common sense, 811 knew that tobacco is one of the causes of oral cancer.

**Conclusions:**

The Egyptian population demonstrated a low knowledge of oral cancer, a finding that is supported by the results of many previous studies conducted on other populations. This finding was significantly influenced by a sociodemographic factor, that is, a higher level of education and young age. Therefore, educational programs and specialized departments are recommended to work on providing and imparting deeper knowledge of oral cancer.

## 1. Introduction

Oral cancer (OC) is defined by the World Health Organization (WHO) as cancer that affects the oral cavity mucosa and tongue (including lip) which ranks as the 16th common cancer globally [1]. New annual cases of OC were estimated at about 354.900 globally with the age standardized rates of 4.0 per a population of 100,000 in 2018 and an annual mortality of around 177.000 of the population [2].

The risk factors of OC include, and are not limited to, smoking, alcohol consumption, smokeless tobacco, betel nut chewing, sunrays, and infection by human papillomavirus (HPV) or Candida. Aging, sunrays, immunosuppression, diet, socioeconomic status, familial and genetic factors, poor diet, and exercise all are risk factors [3, 4]. Worldwide, the most documented risk factor associated with OC is tobacco use [5]. In Egypt, 52% of the detected OC cases were either smokers or former smokers as reported by Attar et al. [6]. These risk factors tend to be poorly perceived or even noticed by the general population [7]. In addition, it has been reported that the majority of certain populations failed to recognize the early signs of OC [4].

Based on the most recent WHO data from 2020, OC accounted for 0.15% of total deaths in Egypt. The death rate from OC is about 1.16 per 100,000 people, placing Egypt at rank number 170 globally [8]. Even though the oral cavity is easily accessible for routine screening and clinical examination, which would enable early diagnosis of malignant alterations, OC is nevertheless one of the most disfiguring and disabling cancers and a highly deadly disease [9, 10]. The significant morbidity and mortality associated with OC are primarily due to the delay in detection rather than the cancer′s aggressiveness [11].

Accordingly, increasing the knowledge and awareness of the signs, symptoms, and early detection methods of OC among the general population can clearly help in its prevention, better treatment, and prognosis. This consolidates the high importance of maintaining community and dental professionals′ efforts in the early detection and prevention of OC by means of outreach programs, integrated educational and media campaigns to raise awareness of the population about OC [12]. Moreover, to conduct effective prevention programs, we need to know the level of OC awareness and knowledge of the population and their interest in learning about OC [13].

Several studies have been conducted globally to evaluate the knowledge and awareness level of the general population regarding OC, but to the best of our knowledge, there is a lack of data regarding the knowledge and the level of awareness of OC among the general population in Egypt. To fill this research gap, the present study is aimed at assessing the level of knowledge and awareness of OC among a general population of Egyptians and at correlating the main factors associated with the level of knowledge.

## 2. Materials and Methods

A face‐to‐face questionnaire based on a cross‐sectional survey was performed on a sample of the general population who do not belong to any health sector. The study was conducted in Cairo, the capital city of Egypt. Before submitting the final questionnaire, a pilot study with primary ethical committee approval in June 2020 from the Research Ethics Committee, Faculty of Oral and Dental Medicine, Ahram Canadian University, Giza, Egypt, was carried out on a random sample of the population (*n* = 35), followed by the modification of the questionnaire based on the pilot study feedback, community standards, and local culture in order to cover the demographic details, knowledge, awareness, and habits of the population. The final questionnaire consisted of 20 questions listed in (Table 1), divided into three main sections: demographic details (4 questions), knowledge and awareness (13 questions), and habits (3 questions), divided as unidirectional or dichotomous questions (yes or no). Only three questions which are generally asked questions contained the answer (do not know). As the questioner was a face‐to‐face type, the research team checked that each participant answered all the questions and signed the consent; hence, there was not any missed data (Supporting Information 1: File S1 and Supporting Information 2: File S2).

**Table 1:** The questionnaire.

Demographic details (4 questions):
1. How old are you?
2. What is your gender identity?
3. What is the highest degree or level of education you have completed?
4. What is your occupation?
Knowledge and awareness (13 questions):
1. Have you heard about oral cancer?
2. Do you know anyone who had oral cancer?
3. Do you know the cause of oral cancer?
4. Can tobacco products cause oral cancer?
5. Can alcohol drinking cause oral cancer?
6. Can any virus cause oral cancer?
7. Can the sun rays cause oral cancer?
8. Is poor oral health a cause of oral cancer?
9. Is oral cancer a contagious disease?
10. Is oral cancer a curable disease?
11. Is oral cancer a preventable disease?
12. Does your healthcare provider educate you about oral cancer?
13. Do you know any symptoms of oral cancer?
Habits (3 questions):
1. Do or did you use tobacco products such as cigarettes, shisha, cigars, e‐cigarettes etc.?
2. If yes, what type of tobacco products do/did you use?
3. Do you drink alcohol?

Ethical clearance, approval, and protocol for the present study were updated and obtained from the Institutional Review Board (No. IRB00012891#130), Faculty of Oral and Dental Medicine, Ahram Canadian University, Giza, Egypt. A well‐structured questionnaire was developed, yet with modifications, based on previously validated items [14, 15] to suit the local population, the culture of Egyptian society, and local risk habits. The questionnaire was conducted in English and then translated into Arabic as it is the mother language of the target population (Supporting Information 1: File S1).

The sample size was calculated based on 80% power, 95% confidence, and a minimum of 1028 participants. Ten public places located at various parts of Cairo were carefully selected to recruit the population from different socioeconomic backgrounds. One thousand twenty‐eight Egyptian participants were present in the selected public places during the period of 15 September to 15 October 2022, and they were verbally informed about the study aim and purpose in detail; participants were only limited to those who understood the purpose of the study and were able to give written consent after reading the information sheet. Also, permissions were obtained from the public places′ managers before conducting the study.

A previous study reported that there is a significant effect of the use of informational flyers on raising the long‐term OC awareness and increasing the knowledge of the general public [16]. Distributed informational flyers after answering the questionnaire for each participant contained a set of clinical characteristics of suspected oral malignant lesions with related pictures.

## 3. Statistical Analysis

Questionnaire data were manually entered into Excel (Microsoft Corporation, Seattle, Washington) and checked by the authors. The data were investigated for normality with the use of Kolmogorov–Smirnov and Shapiro–Wilk tests, showing nonparametric distribution (nonnormal).

Frequencies and percentages followed by chi‐square test were calculated for each question following a scoring system. For the aim of calculating the correlation between the answers of different questions, firstly, a Mann–Whitney test was carried out to compare each two variables in nonrelated samples, and, secondly, a Kruskal–Wallis test to compare more than two variables in nonrelated samples. The significance level was set at *p* ≤ 0.05. This statistical analysis was performed using IBM SPSS Statistics Version 19 for Windows.

## 4. Results

This study covered a total of 1028 participants who fully completed a questionnaire and so were all included in the analysis; all of them were out of the health providers. Of all the participants, 650 (63. 2%) were males and 378 (37.8%) were females and 631 (61.4%) of the participants were in the age group of 18–40 years. As regards the educational level, 647 (62.9%) had a university degree; 405 (39.4%) of the participants were unemployed. A summary of the participants′ sociodemographic data (age, gender, education, and occupation) can be found in (Table 2).

**Table 2:** Summary of the participants′ sociodemographic data (gender, age, education, and occupation).

Variables	Total	Number (%)
Gender	Male	650 (63.2)
	Female	378 (37.8)
Age groups (years)	< 18	87 (8.5)
	18–40	631 (61.4)
	40–70	268 (26.1)
	> 70	42 (4.1)
Education	No formal education	73 (7.1)
	High school	228 (22.2)
	University	647 (62.9)
	Postgraduate	80 (7.8)
Occupation	Private	352 (34.2)
	Public	271 (26.4)
	Unemployed	405 (39.4)

The overall knowledge and awareness of OC in the Egyptian selected population was unsatisfactory, with 40% scoring 6–8 out of the maximum score, that is, 17. In a response to the questionnaire item “have you heard about OC?” only 531 (51.7%) of the total 1028 participants answered yes. Interestingly though, 123 of the participants (12%) knew someone who had OC and only 144 (14%) of the participants said that their healthcare provider educated them about OC.

A clear demarcation was observed in the answers to these questions “is OC a preventable disease?,” “is OC a curable disease?,” and “is OC a contagious disease?” which showed a general misconception (Table 3).

**Table 3:** Generally asked questions.

	Number	%	p‐value
Is oral cancer a contagious disease?			
Yes	242	23.5%	< 0.001 ^∗^
No	531	51.7%	
Do not know	255	24.8%	
Is oral cancer a curable disease?			
Yes	624	60.7%	< 0.001 ^∗^
No	198	19.3%	
Do not know	206	20.0%	
Is oral cancer a preventable disease?			
Yes	801	77.9%	< 0.001 ^∗^
No	212	20.6%	
Do not know	15	1.5%	

^∗^Statistically significant at confidence level 95%.

Regarding the risk factors of OC, only 335 of the participants (32.6%) said that they knew the causes of OC. However, 78.9% (811) knew that tobacco can be a cause of OC and 638 (62.1%) mentioned alcohol as one of the causes of OC. However, regarding their knowledge about the other potential causes of OC, 632 (61.5%) said that they knew that poor oral hygiene could be a risk factor for OC. However, 405 (39.4%) suggested that some viruses may cause OC, and only 229 (22.3%) said that sun rays could be a cause of OC.

In terms of the question related to evaluating the participants′ knowledge about OC symptoms, 485 of the participants (47.2%) did not know the symptoms of OC.

As for the use of tobacco products, 515 of the participants (50.1%) said that they do use or did use tobacco products with no significant difference across the answers (*p* = 0.95). About 29.3% of tobacco users smoke cigarettes and 24.4% smoke waterpipe tobacco. However, 34% of the studied population did not use any tobacco products.

The sociodemographic data were proportional to tobacco use. With regard to the correlation between tobacco use and gender, 408 out of 650 tobacco users (63%) were males and 107 out of the 378 (28%) were females. Among the age groups, the use of tobacco products was highest in the 18–40 age group with 320 users out of a total of 631 given that almost a third of tobacco users had university‐level education (Figures 1a, 1b, and 1c).

Figure 1(a) Correlation between the use of tobacco and sociodemographic data as gender. (b) Correlation between the use of tobacco and sociodemographic data as age. (c) Correlation between the use of tobacco and sociodemographic data as level of education.

**Figure:**
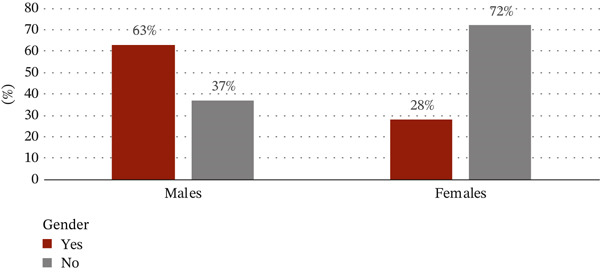
(a)

**Figure:**
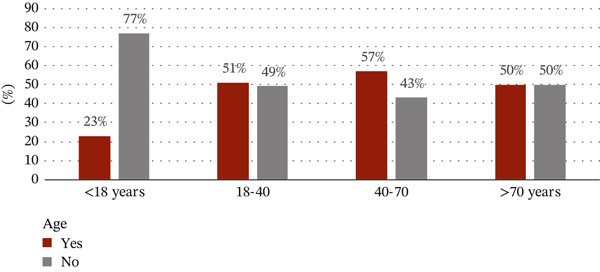
(b)

**Figure:**
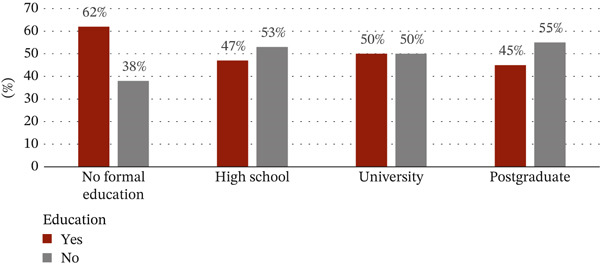
(c)

Only 80 of the 1028 participants (7.8%) answered yes to the questionnaire item “do you drink alcohol?” Sixty‐two out of the 80 participants were males and 18 were females. Fifty‐three of the alcohol consumers were in the 18–40 age group, with 55 having a university degree. In addition, the correlation between habitual tobacco use and habitual alcohol intake showed that there was a significant statistical difference (*p* = 0.011).

The main question that evaluates the general knowledge was cross tabulated with variables such as age, gender, and level of education. Out of a total of 1028 participants, 306 males and 225 females heard about OC. Most of them, 363 (68.3%) out of 531, had a university degree. Almost 70% of them were at the age group of 18–40 years (Figures 2a, 2b, and 2c).

Figure 2(a) Correlation between the question “do you hear about oral cancer?” and sociodemographic data as gender. (b) Correlation between the question “do you hear about oral cancer?” and sociodemographic data as age. (c) Correlation between the question “do you hear about oral cancer?” and sociodemographic data as level of education.

**Figure:**
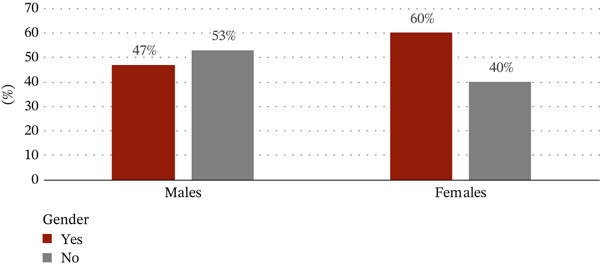
(a)

**Figure:**
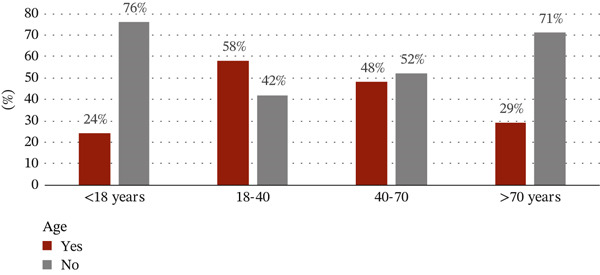
(b)

**Figure:**
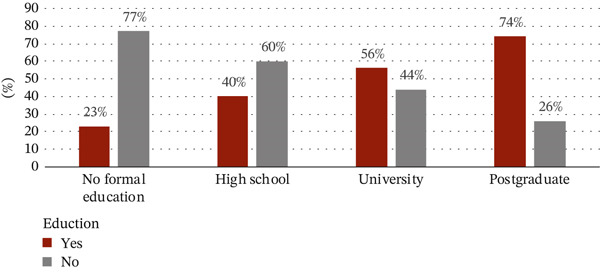
(c)

## 5. Discussion

More sincere efforts should be directed at raising public awareness about OC, its risk factors, signs, and symptoms, which could lead to its early detection, improvement of treatment measures and outcomes, and better survival chances. Therefore, expanding the knowledge of the population about the early signs of cancers will undoubtedly enable patients to be alert and present at an early stage. To the best of our knowledge, this is the first study in Egypt to assess the level of knowledge and awareness of OC among the general population.

Unsurprisingly, the overall awareness level was unsatisfactory in Egypt, especially that a large majority of the Egyptian population lack the basic knowledge about OC. The results found herein are similar and clearly correspond to those found in other studies conducted in Jordan, United States, India, Iran, Saudi Arabia, Yemen, and Spain which showed low level of awareness and knowledge of OCs with the following percentage of the studied population (45.6%, 40%, 35.7%, 32.3%, 72.5%, 53.6%, and 4.3%), respectively [4, 13, 15, 17–20]. In contrast, the level of awareness was high in the United Kingdom, Sri Lanka, Malaysia, China, Australia, and Italy where more than two‐thirds of the studied populations had a good knowledge about OC with the following percentage, respectively, 75%, 62%, 90%, 84.2%, 86.3%, 90%, and 68.4% [21–27].

In regard to the socioeconomic status based on the World Bank country classifications by income level (July 2025–June 2026) [28], Egypt is regarded as a lower middle‐income country, similar to India and Jordan. Based on the previously documented studies compared to ours, Yemen′s economy is low‐income. Iran, on the other hand, is regarded as an upper‐middle‐income country. However, the United States, Saudi Arabia, and Spain are all exhibiting the same low level of OC awareness and knowledge [4, 13, 15, 17–20]. On the other end of the spectrum, countries that are regarded as upper‐middle to high‐income economies, such as the United Kingdom, Sri Lanka, Malaysia, China, Australia, and Italy, have high levels of awareness. That observation points out that the socioeconomic status does not consistently predict a country′s level of OC awareness and knowledge, as studies from the United States, Spain, and Saudi Arabia, which are high‐income countries with low awareness, suggest that other factors are at play, such as oral health systems or cultural gaps (e.g., tobacco use) specific to OC [21–27].

Although in the study conducted in Chine the OC awareness was high, the knowledge about the risk factors and early signs was very low [25]. Disappointingly, our results demonstrate that only 14% of the studied population had heard about OC from a health provider. To bridge this knowledge gap, dentists are required to exert and coordinate more efforts specifically directed at informing the public about OC and its risk factors and signs through media campaigns via the internet, TV, and social media. In regard to the social media benefits in health‐related outcomes, a study reported by Han et al. [29] showed that multiple health benefits can be achieved from social media as it can improve the health communications and cognitive engagement. A recent study conducted by Ria et al. [30] supported the idea of using social media to spread the knowledge and awareness of OC among all group ages.

On the general knowledge questions about whether OC is contagious, curable, or preventable, around 23.5% stated that OC is contagious along with 60% saying that it is curable and 77.9% realizing, based on prior knowledge, that it is preventable; these results were in line with the results of Al‐Maweri et al. [19] and Al‐Maweri et al. [20]. The current study shows that there is a lack of sufficient knowledge about the risk factors of OC. However, almost 79% of the studied population knew that tobacco products could cause OC. On the other hand, 638 of the participants (62.1%) reported that alcohol consumption can cause OC, in reverse to what Shimpi et al. [15] reported. Regarding the role of viruses and sunrays in OC development, the studied population lacked the knowledge of their effect and/or role in OC, a result that was like those of Shimpi et al. [15]. Interestingly, 61.5% of participants in this study knew that personal oral hygiene could be a risk factor for OC.

Based on the findings of our questionnaire, several socioeconomic factors could affect the knowledge of the general population such as age, gender, and level of education. In the current study, there was no statistical significance between males and females′ level of knowledge, a result that was supported by Al‐Maweri et al. [20]. However, this very result contrasts with Ghani et al. [ 24], Hasson et al. [4], and Al‐Maweri et al. [19], who reported that females had better knowledge of OC compared to males based on the females′ tendency to be highly aware of their health.

Regarding the level of awareness, there was a statistically significant difference at the age group of 18–40 years which worked in line with the findings of Hasson et al. [4], Al‐Maweri et al. [19], Ghani et al. [24], and Ria et al. [30]. Such a difference may be attributed to greater exposure to media outlets by younger ages, yet this result conflicts with what was reported by Al‐Maweri et al. [ 20] who did not find any significant difference within the age group. As an indication of research strength, the previous studies [4, 19, 20, 24, 27] reported that 30.6%, 50.5%, 53.6%, 79.9%, and 38.5% of the studied population with university‐level education were highly aware of OC; this consolidates the results of the present study regarding OC knowledge and level of education.

Though the present study has attempted to provide robust answers to the research question, it acknowledges some limitations as well; for example, the time during which the questionnaire was investigated and completed was only a month because of the limited time frame dedicated to graduation project preparation and discussion. In particular, the selected population was not large enough to establish research validity.

Additionally, the study did not assess the source of information regarding the knowledge of participants about OC; in addition, it did not provide an assessment of the frequency and cessation of habits such as smoking or alcohol intake. For that, further studies are encouraged to target a larger population with the use of a more comprehensive questionnaire covering other detailed items.

Despite these limitations, this study has managed to give valuable results, being the first study of its kind to evaluate the level of public awareness and knowledge about OC in Egypt.

## 6. Conclusions

The study gives a clear indication that the Egyptian population has obvious perception problems and wide knowledge gaps about OC. It also substantiates that sociodemographic factors such as a high level of education and younger age play an integral role in determining this level of OC knowledge, a finding that is highly supported by the already existing literature in other countries.

## Author Contributions

Layla Hafed — supervision, conceptualization and methodology, formal analysis, interpreting the results, writing and reviewing the manuscript. Ahmed Abodowia, Mahmoud Elshazly, Mansour El Sayyed, Mariam Elshobaky, Menna‐Allah Fayze and Mina Raafat—data collection and extraction, interpreting the results, writing the manuscript. Gamilah Al‐Qadhi—data analysis, writing review and editing the manuscript. Layla Hafed had full access to all of the data in this study and takes complete responsibility for the integrity of the data and the accuracy of the data analysis.

## Funding

No funding was received for this manuscript.

## Disclosure

All authors have read and approved the final version of the manuscript.

## Ethics Statement

The questionnaire and methodology for this study were approved by the Institutional Review Board (No. IRB00012891#130), Faculty of Oral and Dental Medicine, Ahram Canadian University, Giza, Egypt, and are available upon request.

## Consent

There is no individual person′s data in any form, and the consent of participation and publication of the study was done and approved by the Institutional Review Board (No. IRB00012891#130), Faculty of Oral and Dental Medicine, Ahram Canadian University, Giza, Egypt.

## Conflicts of Interest

The authors declare no conflicts of interest.

## Supporting Information

Additional supporting information can be found online in the Supporting Information section.

## Supporting information


**Supporting Information 1** File S1: The questionnaire included an explanation of the study along with informed consent from the participants.

**Supporting Information 2** File S2: Arabic questionnaire with consent.

## Data Availability

All data in this study are included as supporting information.
