# Supplementary material for: Egyptian General Population Knowledge and Awareness Toward Oral Cancer: A Cross‐Sectional Study
Source: ScientificWorldJournal. 2026 Mar 16;2026:4032372. doi: 10.1155/tswj/4032372 (PMC13140202; doi:10.1155/tswj/4032372)
Supplement: Supplementary file 1 — Supporting Information 1 File S1: The questionnaire included an explanation of the study along with informed consent from the participants. [file TSWJ-2026-4032372-s001.pdf]

Questionnaire for Oral Cancer Awareness
Faculty of Dentistry, Ahram Canadian University
The Egyptian Arabic Republic

You are invited to participate in this survey of oral cancer awareness. We are interested in finding out your knowledge about oral cancers.

Your participation in this study will require participation in a focus survey and possible completion of a questionnaire. This should take approximately 15 minutes of your time. Your participation will be confidential and you will not be contacted again in the future. You will not be paid for being in this study. This focus survey does not involve any foreseeable risk to you and there are no direct benefits. However, the benefits of your participation may impact society by increase the society knowledge about oral cancers. You do not have to be in this study if you do not want to be. But we will be happy by your participation and also to answer any questions you have about this study.

If you have further questions about this project or if you have a research-related problem, you may ask us.

Thank you in advance for you participation in this study.

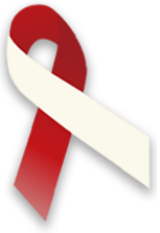

ACU
جامعة الأهرام الكندية
AHRAM CANADIAN UNIVERSITY

Questionnaire for Oral Cancer Awareness

1.How old are you?

☐ < 18 years.
☐ 18-40 years.
☐ 40 – 70 years.
☐ >70 years.

2. What is your gender?

☐ Male.
☐ Female.

3. What is the highest degree of education you have completed?

☐ No schooling.
☐ School.
☐ University.
☐ Postgraduate.

4.What is your occupation?

☐ Private.
☐ Public sector.
☐ Unemployed.

5.Have you heard about oral cancer?

☐ Yes.
☐ No.

6.Do you know anyone who had oral cancer?

☐ Yes.
☐ No.

7.Do you know the cause of oral cancer?

☐ Yes.
☐ No.

8. Is oral cancer a contagious disease?

☐ Yes.
☐ No.
☐ Do not know.

9. Is oral cancer a curable disease?

☐ Yes.
☐ No.
☐ Do not know.

10. Is oral cancer is preventable?

☐ Yes.
☐ No.
☐ Do not know.

11. Can smoking cause oral cancer?

☐ Yes.
☐ No.

12. Can alcohol cause oral cancer?

☐ Yes.
☐ No.

13. Can any virus cause oral cancer?

☐ Yes.
☐ No.

14. Can the sunrays cause oral cancer?

☐ Yes.
☐ No.

15. Is poor oral health a cause of oral cancer?

- ☐ Yes.
- ☐ No.

16. Does your healthcare provider educate you about mouth cancer?

- ☐ Yes
- ☐ No

17. Do you know any symptoms of oral cancer?

- ☐ Non- healing ulcer.
- ☐ Mass.
- ☐ Red patch.
- ☐ White patch.
- ☐ Difficulty in chewing or swallowing.
- ☐ Unexpected weight loss.
- ☐ I do not know any of them.

18. Do or did you smoke tobacco products such as cigarettes, shisha, cigars, e-cigarettes etc.? (If your answer is no move to Q. 20)

- ☐ Yes
- ☐ No

19. If yes, what do you smoke?

- ☐ Cigarettes.
- ☐ Shisha
- ☐ Cigars
- ☐ Pipe
- ☐ E-cigarettes

20. Do you drink alcohol?

- ☐ Yes
- ☐ No

Thank you

I give my consent for participation in the survey on oral cancer. I have answered the above questions to the best of my knowledge and out of my own will.

Name (optional): …………………………………………………............

Signature: ………………………………………………………
