# Supplementary material for: Egyptian General Population Knowledge and Awareness Toward Oral Cancer: A Cross‐Sectional Study
Source: ScientificWorldJournal. 2026 Mar 16;2026:4032372. doi: 10.1155/tswj/4032372 (PMC13140202; doi:10.1155/tswj/4032372)
Supplement: Supplementary file 2 — Supporting Information 2 File S2: Arabic questionnaire with consent. [file TSWJ-2026-4032372-s002.pdf]

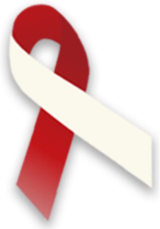

جامعة الأهرام الكندية
ACU
Ahram Canadian University

إستبيان توعية حول سرطان الفم

١. كم عمرك؟

☐ أقل من ١٨ سنة.
☐ بين ١٨ - ٤٠ سنة.
☐ بين ٤٠ – ٧٠ سنة.
☐ أكثر من ٧٠ سنة.

٢. ماهو جنسك؟

☐ ذكر
☐ أنثى

٣. ماهي اعلى درجة علمية حصلت عليها؟

☐ أمي.
☐ مدرسي.
☐ جامعي.
☐ حاصل على درجة في الدراسات عليا (ماجستير، دكتوراه).

٤. ماهي وظيفتك؟

☐ عمل خاص.
☐ قطاع عام.
☐ لا أعمل.

٥. هل سمعت من قبل عن سرطان الفم؟

☐ نعم.
☐ لا.

٦. هل تعرف أحد قد أُصيب من قبل بسرطان الفم؟

☐ نعم.
☐ لا.

٧. هل تعرف أسباب الاصابة بسرطان الفم؟

☐ نعم .
☐ لا.

٨. هل سرطان الفم مرض معدي؟

☐ نعم.
☐ لا.
☐ لا أعلم.

٩. هل يمكن العلاج من سرطان الفم ؟

☐ نعم.
☐ لا.
☐ لا أعلم.

١٠. هل يمكن تجنب الاصابة بسرطان الفم؟

☐ نعم.
☐ لا.

١١. هل تعلم أن التدخين يسبب سرطان الفم؟

☐ نعم.
☐ لا.

١٢. هل تعلم أن شرب الكحوليات يسبب سرطان الفم؟

☐ نعم.
☐ لا.

١٣. هل تعلم أن هناك فيروسات قد تسبب سرطان الفم؟

☐ نعم.
☐ لا.

١٤. هل تعلم أن أشعة الشمس تسبب سرطان الفم؟

☐نعم.
☐ لا.

١٥. هل تعلم أن عدم الاهتمام بصحة الفم قد يسبب سرطان الفم؟

☐ نعم.
☐ لا.

١٦. هل أنت مدخن / كنت مدخن؟

☐نعم.
☐ لا.

١٧. إذا كانت اجابتك نعم عن السؤال السابق، ماذا تُدخن؟

☐ سيجارة.
☐ شيشة.
☐ سيجار.
☐ بايب/ غليون.
☐ سيجارة الكترونية.

١٨. هل تشرب الكحوليات ؟

☐ نعم.
☐ لا.

١٩. هل أخبرك طبيبك من قبل عن سرطان الفم؟

☐ نعم.
☐ لا.

٢٠. هل تعرف أي أعراض لسرطان الفم؟

☐ قُرحة لاتتماثل للشفاء.
☐ كتلة.
☐ بقعة حمراء.
☐ بقعة بيضاء.
☐ صعوبة في المضغ أوالبلع.
☐ نقص غير مبرر في الوزن.
☐ لا اعرف أي منها.

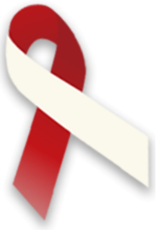

أوافق على المشاركة في الاستبيان الخاص بالتوعية حول سرطان الفم وقد أجبت على الأسئلة أعلاه بحسب معرفتي وبإرادتي الخاصة.
الاسم (اختياري): ....
التوقيع: ....

شكرا لك :)

جامعة الأهرام الكندية
AHRAM CANADIAN UNIVERSITY

إستبيان توعية حول سرطان الفم
كلية طب الأسنان، جامعة الأهرام الكندية
جمهورية مصر العربية

أنت مدعو للمشاركة في هذا الاستطلاع حول سرطان الفم. نحن مهتمون بمعرفة مدى وعيك بمرض سرطان الفم.

سوف يتطلب ذلك مشاركتك في هذه الدراسة وإستكمال هذا الإستبيان. يُتوقع أن يستغرق هذا حوالي ١٥ دقيقة من وقتك. ستكون مشاركتك سرية تماما ولن يتم الاتصال بك في المستقبل. لن يتم الدفع لك مقابل هذه الدراسة. لا تُمثل هذة الدراسة أي خطر متوقع عليك ولا توجد فوائد مباشرة لها. ومع ذلك، قد تؤثر فوائد مشاركتك على المجتمع عن طريق زيادة وعي المجتمع حول سرطان الفم .إذا كنت لا تريد المشاركة، ليس عليك أن تشارك في هذه الدراسة. ولكننا سنكون سعداء بمشاركتك وكذلك بإجابتك على أي أسئلة لديك حول هذه الدراسة.

إذا كانت لديك أسئلة أخرى أو إذا كانت لديك مشكلة متعلقة بالبحث، يمكنك سؤالي.

شكرا لك مقدما على مشاركتك في هذه الدراسة.
